# Non-Targeted Analysis of Per- and Polyfluoroalkyl Substances in Blue Crab

**DOI:** 10.3390/foods15061064

**Published:** 2026-03-18

**Authors:** Maria Nobile, Luca Maria Chiesa, Renato Malandra, Sergio Ghidini, Sara Panseri

**Affiliations:** 1 Department of Veterinary Medicine and Animal Science (DIVAS), University of Milan, 26900 Lodi, Italy; luca.chiesa@unimi.it (L.M.C.); sergio.ghidini@unimi.it (S.G.); sara.panseri@unimi.it (S.P.); 2 ATS Milano-Città Metropolitana, Director of Veterinary Unit, Via Celoria 10, 20133 Milan, Italy; malandra55@libero.it

**Keywords:** blue crabs, PFAS, untargeted analysis, food safety

## Abstract

Per- and polyfluoroalkyl substances (PFASs) are persistent environmental contaminants, yet food safety assessments target only a limited subset despite the presence of numerous, often unidentified, compounds in food matrices. The invasive blue crab (*Callinectes sapidus*), increasingly marketed to reduce ecological and fishery impacts, represents a potential pathway of exposure. As a benthopelagic species, they are prone to accumulate environmental contaminants such as PFASs, so untargeted analysis was carried out by HPLC-HRMS both in the claws and the cephalothorax of 113 blue crabs from the Adriatic Sea (FAO zone 37.2.1). The results revealed the presence of five suspected compounds (1,2,2,3,3,4,5,5,6-nonafluoro-4,6,bis(trifluomethyl)cyclohexane-1-sulfonic acid, VUNTWVAXULT-MOZ-UHFFFAOYSA-N, 1-hydro-pentadecafluoroheptane, 1H-perfluorohexane, SPSZZAMPELOEOG-UHFFFAOYSA-N) other than common PFASs, which have already been fully investigated in our previous work, along with 10 proposed PFOA isomers as tentative candidates. Hierarchical clustering analysis showed distinct patterns of distribution between tissue types in the samples with higher concentrations. All suspected molecules, except one, are found in higher amounts in the claws, which are the parts monitored for legacy PFASs by the current regulation. These findings could improve risk assessment for their potential implications on human health and food safety, as well as have possible significance on toxicological, environmental and regulatory relevance.

## 1. Introduction

Per- and polyfluoroalkyl substances (PFASs) are persistent environmental contaminants with significant bioaccumulative potential, raising growing public health concerns [1]. Due to their extensive industrial and commercial use, they are now widespread across ecosystems worldwide. Industrial uses include anti-corrosive and water-repellent coatings, lubricants and hydraulic fluids in the aerospace and automotive sectors; firefighting foams at airports, military sites, and industrial facilities; and oil- and water-resistant coatings in the production of paper and cardboard. Commercial and consumer applications encompass non-stick cookware, stain- and water-repellent textiles and carpets, waterproof clothing and outdoor gear, cosmetics and personal care products, packaging materials such as fast-food wrappers and microwave popcorn bags, and electronic components requiring insulating or hydrophobic materials. Regulatory actions have banned or restricted several PFASs, such as PFOS (perfluorooctanesulfonic acid) and PFOA (perfluorooctanoic acid), owing to their toxicity [2].

Despite these efforts, current food safety risk assessments focus on a limited number of well-known target PFAS compounds, mostly perfluoroalkyl acids (PFAAs) (e.g., PFOS, PFOA), with approximately 30 compounds routinely monitored [1], likely underestimating true exposure and health risks. In reality, thousands of PFASs, including replacement chemicals and transformation products, remain unidentified or insufficiently characterized [3]. Untargeted analysis and omics approaches have emerged as promising tools to improve chemical risk assessment and support precautionary regulations [4,5], and similar strategies have been applied to other contaminants [6,7,8].

Monitoring both known and unknown PFASs in the food chain is crucial. The inclusion of untargeted approaches allows a more comprehensive estimate of dietary exposure, supporting mixture-based risk assessments and more conservative regulatory limits that account for potential additive or synergistic effects. Early identification of untargeted PFASs also provides baseline data to inform future regulatory decisions as toxicological information becomes available.

Benthic and benthopelagic species, such as the blue crab (*Callinectes sapidus*), are prone to PFAS bioaccumulation due to their ecological niche and feeding behavior [9,10]. Studies have documented PFAS contamination in the Mediterranean Sea and its biota [11], with significant levels in coastal waters and shellfish. In Lake Ontario, hundreds of unknown PFAS features appear to biomagnify in benthic organisms, suggesting substantial fluorinated contamination [1]. Our previous targeted analysis of 113 Adriatic Sea blue crabs detected 17 PFASs [12], with higher concentrations in the cephalothorax than claws, likely due to PFAS affinity for protein-rich tissues such as liver and hepatopancreas [13]. Consumer exposure may be higher when consuming these edible parts.

Nonetheless, these findings likely represent only a portion of the true PFAS burden. Many unknown or novel PFASs may go undetected with standard targeted methods. To address this gap, non-targeted analytical approaches (NTA) are essential. Techniques such as high-resolution mass spectrometry (HRMS), including QTOF-HRMS and Orbitrap-HRMS, are increasingly employed for the detection of previously unidentified PFASs in food matrices [14,15,16]. Considering the increased market availability and consumption of blue crab in Europe due to its invasive status and efforts to integrate it into the food supply chain [17,18], ensuring comprehensive PFAS monitoring is critical. This study aims to explore the presence of unknown PFASs in blue crab through an untargeted analytical approach, ultimately contributing to more robust food safety risk assessments.

## 2. Materials and Methods

### 2.1. Chemicals and Reagents

All LC–MS-grade solvents and reagents used during the analytical protocol were purchased from Merck (Darmstadt, Germany). The purification cartridges, Strata PFAS (WAX/GCB) 200 mg/50 mg/6 mL, were obtained from Phenomenex (Torrance, CA, USA). The 2 internal standards, octanesulfonic acid (MPFOS) and perfluoro-[1,2,3,4,5–13C5] nonanoic acid (MPFNA), in single stock solutions at a concentration of 50 μg mL^−1^ in methanol were purchased from Chemical Research 2000Srl (Rome, Italy) and diluted to a concentration of 1 μg mL^−1^ before the analytical session, then stored at −20 °C.

### 2.2. Sample Collection

A total of 113 crabs (43 males and 70 females) were obtained from the Milan fish market, originating from the Adriatic Sea (FAO fishing zone 37.2.1). The specimens were collected post-commercial harvest and were not alive at the time of purchase. No animals were sacrificed specifically for the purposes of this study. As the specimens were collected postmortem as part of the normal food supply chain, ethical approval for animal experimentation was not required.

To assess the distribution of per- and polyfluoroalkyl substances (PFASs) across edible body regions, each specimen’s claws and cephalothorax (only the soft tissues, such as muscle and hepatopancreas) were separated and homogenized individually. All samples were stored at −20 °C prior to chemical extraction.

### 2.3. Sample Extraction Protocol

The extraction process was adapted from the method described by Chiesa et al. [19]. Specifically, 5 g of homogenized tissue spiked with internal standards to achieve a final concentration of 2 ng g^−1^ was extracted with 10 mL of acetonitrile. This was followed by 1 min of vortexing and 15 min of sonication. The mixture was then centrifuged at 2500× *g* for 10 min at 4 °C. The resulting supernatant was evaporated and redissolved in 5 mL of water for further purification using STRATA PFAS solid-phase extraction (SPE) cartridges under vacuum. The cartridges were preconditioned sequentially with 4 mL of 0.3% ammonium hydroxide in methanol, 4 mL of pure methanol, and 4 mL of ultrapure water. Following sample application, two washing steps were performed using 2 × 4 mL of water. Analytes were eluted using 2 × 4 mL of 0.3% ammonium hydroxide in methanol. The eluates were dried, then reconstituted in 200 μL of 20 mM ammonium formate: methanol (20:80, *v*/*v*). If turbidity was observed, samples were centrifuged in Eppendorf for 2 min before analysis by high-performance liquid chromatography coupled with high-resolution mass spectrometry (HPLC-HRMS).

### 2.4. HPLC-HRMS Analysis

The HPLC-HRMS analysis was conducted using a Vanquish binary pump coupled with a Thermo Orbitrap Exploris 120 mass spectrometer (Thermo Fisher Scientific, Waltham, MA, USA). Chromatographic separation was performed on a Raptor ARC-18 column (5 μm, 120 × 2.1 mm; Restek, Bellefonte, PA, USA). To delay system-derived PFASs and reduce background interference, a Megabond WR C18 guard column (5 cm × 4.6 mm) was installed upstream of the injector. Stainless steel tubing and components were utilized to further minimize PFAS contamination. The mobile phase consisted of 20 mM ammonium formate in water (phase A) and methanol (phase B). A gradient program began with 20% B and was gradually increased to 95% by minute 7, held for 3 min, and returned to the starting condition by minute 11. This condition was maintained for another 4 min, totaling a 15 min runtime at a flow rate of 0.3 mL min^−1^. Mass spectrometry parameters were set as follows: capillary temperature at 330 °C, vaporizer temperature at 280 °C, sheath gas flow rate of 35 units, and auxiliary gas at 15 units. Electrospray ionization was performed in negative mode at 3.50 kV. The RF lens setting was 70%, with a standard automatic gain control (AGC) and automatic maximum injection time. Two acquisition modes were applied: Full Scan (FS) and Full Scan Data-Dependent MS^2^ (FS-dd-MS^2^). FS mode operated at a resolution of 120,000 (*m*/*z* range: 200–1100), with a 70% RF lens, a normalized AGC target of 100%, and auto injection timing. FS-dd-MS2 was used for targeted fragmentation of precursor ions from FS scans. It featured a resolution of 30,000, an *m*/*z* range of 200–1100, an isolation window of 1.5 *m*/*z*, and stepped normalized collision energies of 10, 35, and 70 eV. The AGC target was set to 100% with a maximum injection time of 85 ms. A dynamic exclusion window of 2.5 s and an intensity threshold of 9.00 × 10^4^ were applied, with a mass tolerance of 5 ppm.

The AcquireX Deep Scan workflow (Thermo Scientific, San Jose, CA, USA) was used to enhance identification and reduce background interference. Procedural blanks were analyzed to generate an exclusion list of background ions, while pooled quality control (QC) samples provided inclusion lists. The software dynamically updated these lists between each injection. The [M−H]^−^ ion was designated as the preferred precursor, and isotope detection was enabled. During initial MS^1^ surveys, no MS^2^ acquisition was performed. Four replicate injections of QC samples were then analyzed using the FS-dd-MS^2^ method to collect MS^2^ spectra (ID, Identification Only files) for compound identification. After each injection, newly identified ions were added to the exclusion list to avoid redundant fragmentation in subsequent runs until all inclusion list ions are fragmented. This workflow was carried out in negative ion mode. Following the completion of AcquireX scanning, all experimental samples were analyzed using the full MS method, with QC samples inserted every six runs to monitor instrument performance. Data acquisition and system control were managed using Xcalibur™ software version 4.5 (Thermo Fisher Scientific, Waltham, MA, USA).

### 2.5. Untargeted Approach by Compound Discoverer™ Workflow

Compound Discoverer™ (CD) version 3.3 SP3 (Thermo Fisher Scientific, Waltham, MA, USA) was employed to process the raw data generated from procedural blanks, identification (ID) files, quality controls (QCs), and crab tissue samples acquired using the Orbitrap Exploris 120 system. The primary objective was untargeted identification of PFASs and subsequent statistical evaluation. The analysis followed the PFAS-specific workflow, a modular arrangement of interconnected processing nodes optimized for PFAS detection (Figure S1). This workflow enabled selection of relevant spectra, alignment of retention times, normalization of peak areas, differentiation of background signals, and compound identification through spectral library matching, alongside optional statistical evaluation [20]. The software utilized procedural blank data to detect background ions not previously excluded by the AcquireX method. QC samples were used to align retention times across the dataset and to facilitate normalization. MS2 spectra from the ID files were extracted and mapped to their corresponding Full Scan (FS) precursors based on retention time and parent *m*/*z* tolerances. These were then compared against spectral databases such as mzCloud™, ChemSpider, and mzVault for compound identification.

In addition to mzCloud’s data-dependent MS2 reference spectra, the workflow included the PFAS_CFM_specLibrary_Duke fn:2 (a predicted spectral library for PFASs), the EPA DSSTox dataset (via ChemSpider for structural and formula-based matching), and multiple PFAS suspect screening lists (PFAS_NEG, PFAS_NIST, and PFAS_SUSPECTDB_DUKE). Advanced analytical features derived from literature—such as mass defect filters tailored to fluorinated compounds, transformation rules, and Kendrick mass defect (KMD) analysis—were incorporated to improve identification accuracy. Fluorinated molecules typically exhibit a negative mass defect, which was exploited using two mass defect strategies: standard and Kendrick. The standard approach calculates the difference between exact and nominal mass to identify homologous series with similar mass defects, while the Kendrick method normalizes repeating units to highlight patterns among chemically related compounds [21]. CD also identified unknowns by recognizing consistent fragmentation patterns using the Compound Class Scoring node. This was based on custom libraries such as PFAS General from FluoroMatch Suite.cLib and PFAS Fine signature fragment_lib.cLib. Logical filtering rules were applied to refine the results. All the following conditions (AND logic) had to be met: a standard mass defect between 0.116 and 0.268 (type: Standard MD), a peak rating above 3.0 in any sample, a class coverage score ≥ 0.25 within the compound class PFAS General from FluoroMatch Suite, the exclusion of background signals, and predicted formulas containing fluorine atoms. Additionally, at least one of these (OR logic) ahad to be true: a best match score > 75 in mzCloud, class coverage > 0 in Compound Class PFAS Fine Signature Fragment_Lib, or a best similarity match in mzCloud > 75. Final confirmation was limited to features with corresponding MS2 spectra (from DDA scans of selected precursors). For cases where database matching failed, manual interpretation of the fragmentation pattern was conducted using ChemDraw Professional version 25.5.0.5789.

### 2.6. Statistical Analysis

Descriptive, univariate, and multivariate statistical analyses were executed as an integral part of the Compound Discoverer workflow by the Differential Analysis node. Hierarchical cluster analysis (HCA) with heat maps and box-and-whisker charts (BWC) were employed to explore the dataset and highlight trends in individual metabolite levels. One-way ANOVA followed by Tukey’s post hoc test was applied to assess statistical differences across groups in the claws vs. cephalothorax comparison. HCA provided insights into relationships among metabolites across samples, represented in a two-dimensional heat map where each cell corresponds to the relative abundance of a specific compound. The clustering followed a bottom-up agglomerative approach, successively merging the most similar clusters and ultimately forming a dendrogram that reflects hierarchical relationships. Box-and-whisker plots displayed distributional statistics including medians, interquartile ranges, and extreme values for each selected metabolite across experimental groups.

## 3. Results and Discussion

From a dataset comprising 7788 detected features, a combination of experimentally measured *m*/*z* values, predicted molecular formulas, isotopic distribution patterns, online spectral matching, and MS2 fragmentation data led to the tentative identification of five suspect compounds not previously described in our earlier PFAS investigations on targeted analysis [12]. To assign confidence levels to these annotations (Table 1), the framework established by Schymanski et al. [22] was employed. All five compounds were classified as Level 2a, indicating that a specific structure could be proposed with high confidence due to a strong and direct match between observed MS2 spectra and those found in reference libraries or published sources. Special care was taken to ensure comparability of spectra by accounting for differences in instrument settings such as resolution, ionization mode, and collision energy. In addition, mzVault analysis suggested the presence of ten isomeric forms of perfluorooctanoic acid (PFOA). These identifications were assigned a Level 3 confidence, meaning that the structural assignment was plausible based on spectral data, but multiple isomers fit the observed patterns [22]. It is desirable for future work to target isolation and full chemical characterization. Detailed attributes—including molecular formulas, proposed names, exact *m*/*z* values, mass errors, diagnostic DDA ions, retention times, peak areas, and confidence levels—are compiled in Table 1.

**Figure 1:**
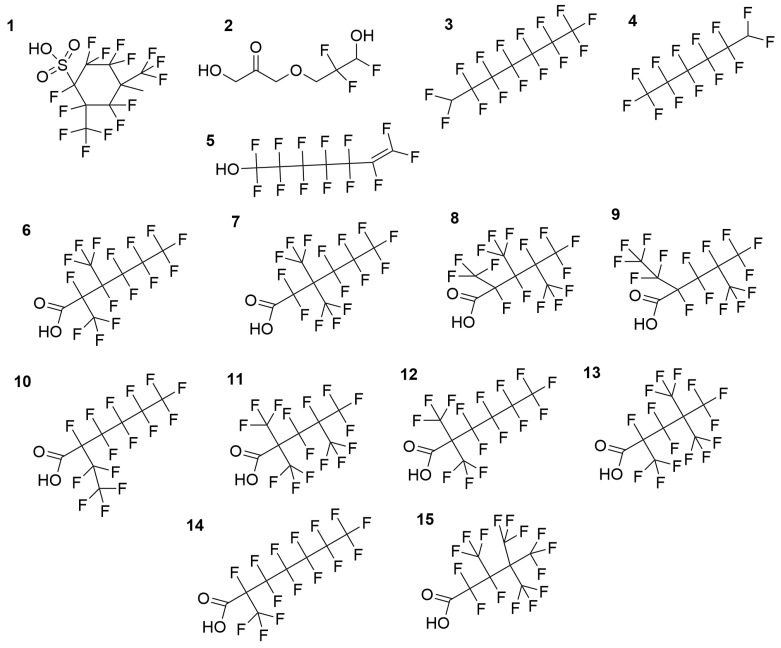
Structures of the 5 suspected untargeted compounds and the 10 PFOA isomers (6–15) proposed by mzVault results.

**Figure 2:**
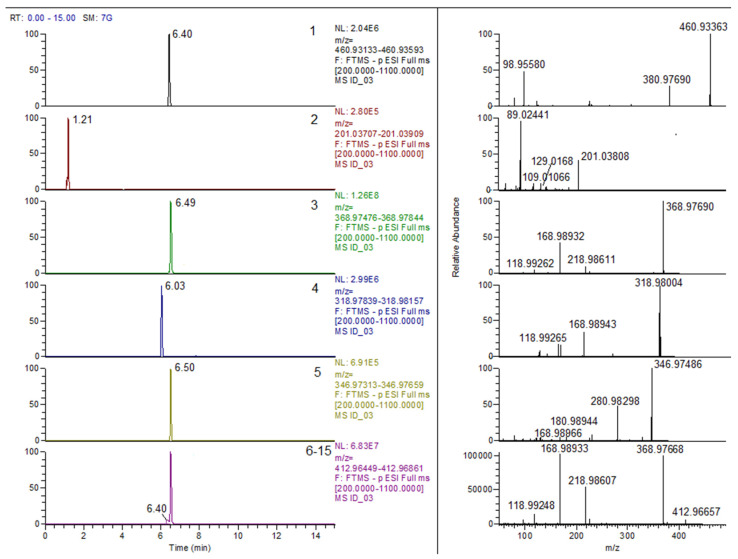
Extracted parent ion chromatograms from FS with the relative fragmentation mass spectra of the untargeted compounds reported in Table 1 from an identification-only crab sample (ID file).

The proposed molecular structures for these compounds are illustrated in Figure 1, while the extracted parent ion chromatograms from FS with the relative fragmentation mass spectra are reported in Figure 2.

**Figure 3:**
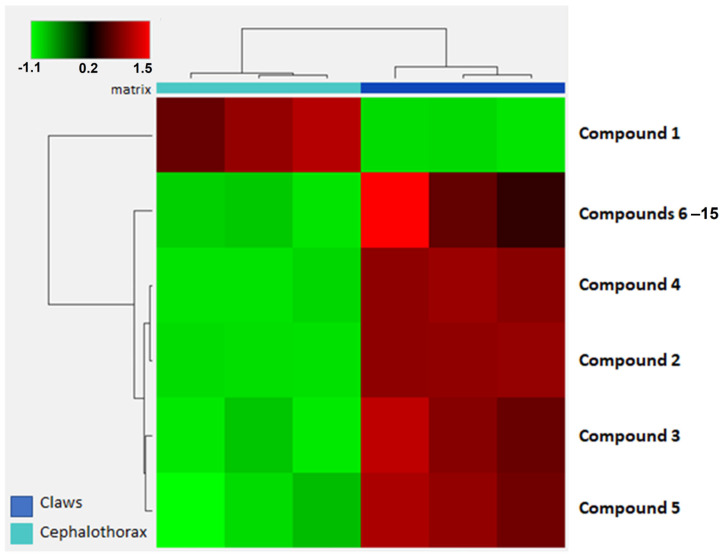
Hierarchical cluster analysis: heat map with the variations between PFASs discovered by the untargeted analysis of the claws and cephalothorax of the 3 male crabs with the highest amount.

Interestingly, both 1-hydro-pentadecafluoroheptane and 1H-perfluorohexane were also detected in a non-targeted PFAS screening of roe deer (Capreolus capreolus) liver and muscle tissues reported by Pavlovic et al. [23]. Furthermore, 1H-perfluorohexane and other perfluoroalkane-type compounds have recently been proposed as novel degradation products resulting from aerobic biotransformation of 8:2 fluorotelomer alcohol (8:2 FTOH) [24]. These putative, untargeted compounds appeared predominantly in male crab samples; however, our data do not allow statistical confirmation of this trend. This pattern may be related to body size and weight as observed for the legacy PFASs in our previous work [12], sex-specific physiological factors, or a combination of both, and thus warrants further investigation. We analyzed the three individuals exhibiting the highest numbers and concentrations of newly detected compounds, aiming to identify the crab edible part in which these compounds were most prominently accumulated, and hierarchical clustering analysis (HCA) via heat map visualization revealed distinct clustering patterns between the cephalothorax and claw samples (Figure 3). This heat map was constructed to illustrate the relative abundance patterns of the five suspect PFASs (Table 1), with compounds showing elevated amounts highlighted in red, and those with lower abundance in green (Figure 3); all suspected molecules, except for compound 1, are found in higher amounts in the claws, which are the parts monitored for legacy PFASs by the current regulation [25].

For the newly identified compounds, it is currently not possible to assess their toxicological or environmental relevance due to the lack of available information. Nevertheless, these findings may serve as a basis for future targeted studies on this subject.

### PFOA Isomers: Environmental Fate, Toxicokinetics and Food Safety Implications

Many unregulated PFASs can act as precursors to PFOA, often containing non-fluorinated segments prone to oxidative degradation while the perfluorinated chain remains resistant [26]. Such transformations have been observed in wastewater treatment, where effluents may contain higher PFAS concentrations than influents. Examples include cationic fluorinated surfactants like perfluorooctane-amido and sulfonamido quaternary ammonium salts [27]. Although the formation of branched PFOA from precursors is not documented, it is plausible and may lead to underestimation in environmental samples [26]. Regulatory limits typically consider linear and branched PFOA together, yet branched forms are often detected in notable proportions, sometimes exceeding linear concentrations due to higher polarity [28]. While branched PFOS has been more studied, branched PFOA has received less attention, partly due to historical manufacturing via electrochemical fluorination and telomerization [27,29]. Structural differences between linear and branched isomers influence persistence, degradation, and toxicokinetics; branched PFOA exhibits shorter biological half-lives due to enhanced renal elimination and lower protein-binding, but can activate PPARα similarly to linear PFOA, raising concerns about hepatic effects, lipid metabolism, and tumorigenesis [27,28,29,30,31].

The lack of commercially available analytical standards for many branched isomers poses a challenge for their toxicological characterization, and data on isomer-specific health effects remain limited. With respect to environmental fate and behavior, branched PFOA isomers demonstrate slightly higher water solubility and lower surface tension, which contribute to their increased mobility in groundwater and surface water systems [32]. This can lead to widespread environmental dispersion. Environmental monitoring studies have shown varying isomer profiles across environmental matrices, with branched isomers often more prevalent in aqueous phases, while linear forms are more likely to accumulate in sediments and organisms [33,34]. Though branched isomers may exhibit slightly faster degradation under some conditions, both linear and branched PFOA are extremely persistent [35]. Their detection in remote regions, including Arctic wildlife, supports their long-range transport potential [36]. Regarding food safety implications, the presence of branched PFOA isomers in the food chain arises from environmental contamination (e.g., irrigation with polluted water), bioaccumulation in livestock, and migration from food-contact materials [2]. Due to limitations in routine analytical methods, isomer-specific quantification is not widely performed, despite evidence that branched and linear PFOA distribute differently in animal tissues and fluids [37]. Current tolerable weekly intake (TWI) values set by EFSA are based on total PFOA concentrations, with minimal differentiation between isomers. However, considering the distinct biological behavior of branched isomers, future food safety assessments may need to incorporate isomer-specific data for more accurate exposure and risk evaluations.

## 4. Conclusions

The presented untargeted study on blue crabs from the Adriatic Sea permitted the successful individuation of five PFASs, different from the common ones, which was supported by strong spectral matches to reference libraries but not confirmed by authentic standards. Moreover, 10 isomers of PFOA were proposed by mzVault results as tentative candidates, with insufficient evidence to assign one exact structure. Non-targeted analytical methods have the potential to significantly transform seafood risk assessment by broadening the scope of detectable contaminants beyond the limited set of known compounds traditionally targeted in monitoring programs, uncovering a much wider range of these substances, including novel or emerging PFASs that may not yet be regulated or even fully characterized. The use of non-targeted approaches highlights the limitations of current seafood safety policies that focus on a narrow list of known PFAS. As new PFAS are detected, some with potentially high bioaccumulation or toxicity, there is an urgent need to reconsider and expand regulatory frameworks. This could lead to more precautionary and comprehensive guidelines for seafood safety, especially for populations with high seafood consumption. A key challenge with non-targeted methods is that detection alone does not imply toxicological relevance. Therefore, there is a critical need to link non-targeted PFAS findings to toxicological studies through structure–activity relationship modeling, bioassays, or prioritization frameworks. This linkage would help assess the actual health risks associated with newly detected PFAS and inform risk-based decision-making. These considerations highlight that current PFAS risk assessments, based on a limited number of known molecules, may underestimate the relative risk for seafood consumers. Monitoring bioaccumulation and identifying new per- and polyfluoroalkyl substances are essential for a comprehensive, One Health–oriented perspective. Non-targeted and suspect screening analyses are powerful tools that warrant further investigation and are expected to play an increasingly important role in exposomic research.

## Figures and Tables

**Table 1:** The molecular formulas, putative names, *m*/*z* value, mass error, diagnostic ions in DDA, retention times (RT), area max and confidence level of the compounds detected by untargeted analysis.

Code	Name	Formula	*m*/*z* [M–H]^−^	Mass Error	Diagnostic Ion in DDA	RT	Area Max	Confidence Level
1	1,2,2,3,3,4,5,5,6-nonafluoro-4,6,bis(trifluomethyl)cyclohexane-1-sulfonic acid	C_8_HF_15_O_3_S	460.93363	0.48	98.95580, 380.97690	6.40	19,750,248	2a
2	VUNTWVAXULT-MOZ-UHFFFAOYSA-N	C_6_H_9_F_3_O_4_	201.03808	0.32	89.02441, 109.01066 129.01689	1.21	3,594,268	2a
3	1-hydro-pentadecafluoroheptane	C_7_HF_15_	368.97660	0.02	118.99262, 168.98932, 218.98611	6.49	801,978,695	2a
4	1H-perfluorohexane	C_6_HF_13_	318.97998	0.59	118.99265, 168.98943	6.03	44,659,697	2a
5	SPSZZAMPELOEOG-UHFFFAOYSA-N	C_7_HF_13_O	346.97486	0.45	168.98966, 180.98944, 280.98298	6.50	5,987,729	2a
**10 PFOA isomers by mzVault**								
6	2,3,4,4,5,5,6,6,6-nonafluoro-2,3-bis(trifluoromethyl)hexanoic acid	C_8_HF_15_O_2_	412.96655	0.27	118.99248 168.98933, 218.98607 368.97668	6.40	8,828,067	3
7	2,2,4,4,5,5,6,6,6-nonafluoro-3,3-bis(trifluoromethyl)hexanoic acid	C_8_HF_15_O_2_	412.96655	0.27	118.99248 168.98933, 218.98607 368.97668	6.40	8,828,067	3
8	2,3,4,5,5,5-hexafluoro-2,3,4-tris(trifluoromethyl)pentanoic acid	C_8_HF_15_O_2_	412.96655	0.27	118.99248 168.98933, 218.98607 368.97668	6.40	8,828,067	3
9	2,3,3,4,5,5,5-heptafluoro-2-(pentafluoroethyl)-4-(trifluoromethyl)pentanoic acid	C_8_HF_15_O_2_	412.96655	0.27	118.99248 168.98933, 218.98607 368.97668	6.40	8,828,067	3
10	2,3,3,4,4,5,5,6,6,6-decafluoro-2-(pentafluoroethyl)-4-(trifluoromethyl)hexanoic acid	C_8_HF_15_O_2_	412.96655	0.27	118.99248 168.98933, 218.98607 368.97668	6.40	8,828,067	3
11	3,3,4,5,5,5-hexafluoro-2,2,4-tris(trifluoromethyl)pentanoic acid	C_8_HF_15_O_2_	412.96655	0.27	118.99248 168.98933, 218.98607 368.97668	6.40	8,828,067	3
12	3,3,4,4,5,5,6,6,6-nonafluoro-2,2-bis(trifluoromethyl)hexanoic acid	C_8_HF_15_O_2_	412.96655	0.27	118.99248 168.98933, 218.98607 368.97668	6.40	8,828,067	3
13	2,3,3,5,5,5-hexafluoro-2,4,4-tris(trifluoromethyl)pentanoic acid	C_8_HF_15_O_2_	412.96655	0.27	118.99248 168.98933, 218.98607 368.97668	6.40	8,828,067	3
14	2,3,3,4,4,5,5,6,6,7,7,7-dodecafluoro-2(trifluoromethyl)heptanoic acid	C_8_HF_15_O_2_	412.96655	0.27	118.99248 168.98933, 218.98607 368.97668	6.40	8,828,067	3
15	2,2,3,5,5,5-hexafluoro-3,4,4-tris(trifluoromethyl)pentanoic acid	C_8_HF_15_O_2_	412.96655	0.27	118.99248 168.98933, 218.98607 368.97668	6.40	8,828,067	3

## Data Availability

The original contributions presented in the study are included in the article/Supplementary Materials. Further inquiries can be directed to the corresponding author.

## Supplementary Materials

The following supporting information can be downloaded at: https://www.mdpi.com/article/10.3390/foods15061064/s1, Figure S1: Workflow used in Compound Discoverer (CD) 3.3 SP3 program.
