# Supplementary figures and images for: Non-Targeted Analysis of Per- and Polyfluoroalkyl Substances in Blue Crab
Source: Foods. 2026 Mar 18;15(6):1064. doi: 10.3390/foods15061064 (PMC13025805; doi:10.3390/foods15061064)

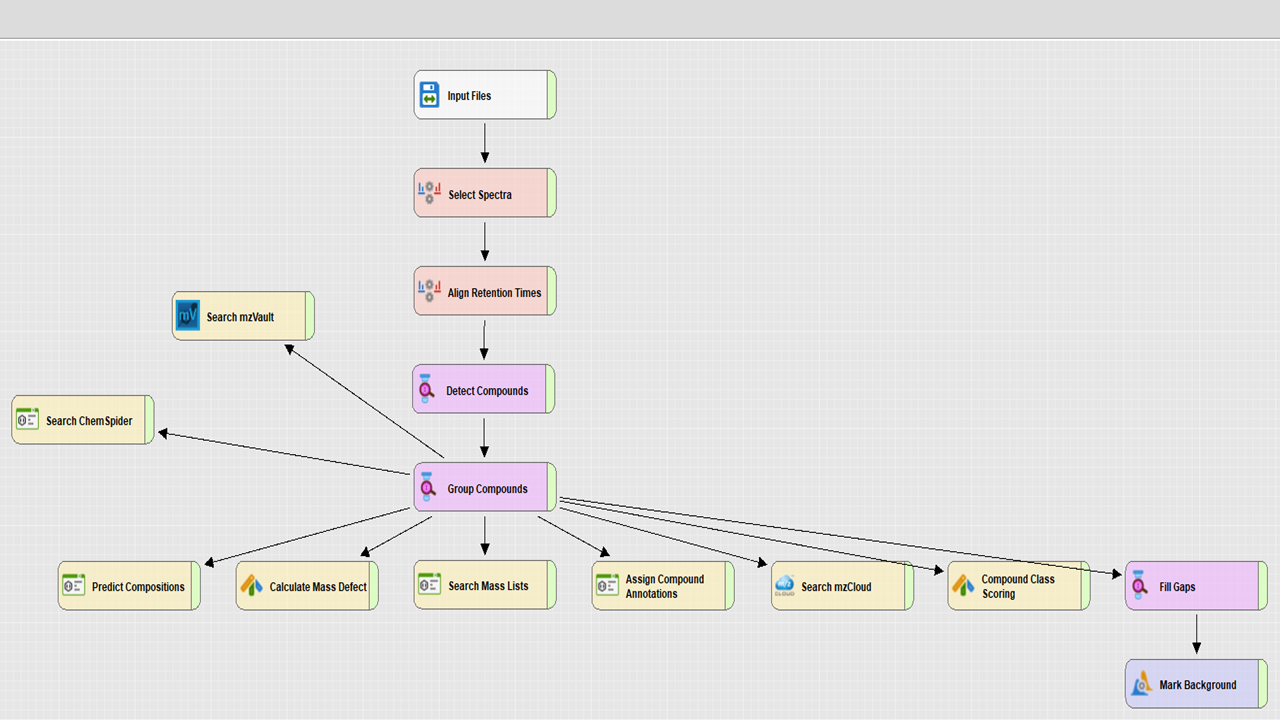

Supplement: Supplementary file 1 [file foods-15-01064-s001.zip › foods-4175168-supplementary.tif]
